# Multivariate meta‐analysis of prognostic factor studies with multiple cut‐points and/or methods of measurement

**DOI:** 10.1002/sim.6493

**Published:** 2015-04-29

**Authors:** Richard D. Riley, Eleni G. Elia, Gemma Malin, Karla Hemming, Malcolm P. Price

**Affiliations:** ^1^ Research Institute of Primary Care and Health SciencesKeele UniversityStaffordshireST5 5BGU.K.; ^2^ School of Health and Population SciencesPublic Health Building, University of BirminghamEdgbaston BirminghamB15 2TTU.K.; ^3^ School of MedicineD Floor Queen's Medical CentreUniversity of NottinghamNottinghamNG7 2UHU.K.

**Keywords:** multivariate meta‐analysis, prognostic factors, odds ratios and hazard ratios, cut‐points, heterogeneity

## Abstract

A prognostic factor is any measure that is associated with the risk of future health outcomes in those with existing disease. Often, the prognostic ability of a factor is evaluated in multiple studies. However, meta‐analysis is difficult because primary studies often use different methods of measurement and/or different cut‐points to dichotomise continuous factors into ‘high’ and ‘low’ groups; selective reporting is also common. We illustrate how multivariate random effects meta‐analysis models can accommodate multiple prognostic effect estimates from the same study, relating to multiple cut‐points and/or methods of measurement. The models account for within‐study and between‐study correlations, which utilises more information and reduces the impact of unreported cut‐points and/or measurement methods in some studies. The applicability of the approach is improved with individual participant data and by assuming a functional relationship between prognostic effect and cut‐point to reduce the number of unknown parameters. The models provide important inferential results for each cut‐point and method of measurement, including the summary prognostic effect, the between‐study variance and a 95% prediction interval for the prognostic effect in new populations. Two applications are presented. The first reveals that, in a multivariate meta‐analysis using published results, the Apgar score is prognostic of neonatal mortality but effect sizes are smaller at most cut‐points than previously thought. In the second, a multivariate meta‐analysis of two methods of measurement provides weak evidence that microvessel density is prognostic of mortality in lung cancer, even when individual participant data are available so that a continuous prognostic trend is examined (rather than cut‐points). © 2015 The Authors. *Statistics in Medicine* Published by John Wiley & Sons Ltd.

## 1 Introduction

A prognostic factor is any measure that, among people with a given health condition, is associated with a subsequent clinical outcome 1, 2. For example, in many cancers, tumour grade at the time of histological diagnosis is a prognostic factor because it is associated with time to disease recurrence or death; those with a higher tumour grade have a worse prognosis. Prognostic factors thus distinguish groups of people with a different average prognosis, and this allows them to be useful for clinical practice and health research. For example, they can help define disease at diagnosis, inform clinical and therapeutic decisions (either directly or as part of multivariable prognostic models), enhance the design and analysis of intervention trials and observational studies (as they are potential confounders) and may even identify targets for new interventions that aim to modify the course of a disease or health condition.

Given their importance, there are often hundreds of studies each year investigating the prognostic value of one or more bespoke factors in each disease field. However, there is often inconsistency in their findings, with some suggesting a particular factor is prognostic and others suggesting the opposite 1, 3, 4. Meta‐analysis is therefore needed to synthesise study findings and summarise the prognostic value of each factor of interest 5. Unfortunately, this is often problematic as primary studies are prone to poor and selective reporting 6, 7, 8 and heterogeneity in, for example, their study populations and type of statistical results 9. Meta‐analyses of prognostic factor studies thus often conclude without strong recommendations 1, 3. The following is a typical example 10: ‘After 10years of research, evidence is not sufficient to conclude whether changes in P53 act as markers of outcome in patients with bladder cancer …. That a decade of research on P53 and bladder cancer has not placed us in a better position to draw conclusions relevant to the clinical management of patients is frustrating.’

Two common problems for meta‐analysis of a particular factor are between‐study differences in its method of measurement and, for continuous factors, the cut‐point value used to define ‘high’ and ‘low’ (or abnormal and normal) groups. For example, de Azambuja *et al.* 11 perform a meta‐analysis of the prognostic ability of Ki‐67 in patients with breast cancer and pool 38 unadjusted hazard ratios across studies; however, these related to 20 different cut‐points and five different methods of measurement. When pooling such studies, the summary meta‐analysis results are difficult to interpret clinically, as they do not relate to a single cut‐point or measurement method. Even if studies do report results for multiple cut‐points or methods of measurement, meta‐analysts usually just take one cut‐point and one method of measurement per study and thus lose information about the others. This may be because multiple study results for each cut‐point and method of measurement are correlated, and therefore, more advanced statistical methods are necessary to account for this if they are all used in the meta‐analysis 12.

In this article, we suggest approaches to meta‐analysis of prognostic factor studies when faced with multiple cut‐points and/or methods of measurement and missing results in some studies. Firstly, in Section 2, we consider methods for situations where each study provides a single prognostic result for a particular cut‐point and method of measurement, but there are between‐study differences in the cut‐point and method of measurement chosen. We show how a 95% prediction interval best summarises a random‐effects meta‐analysis in this situation 13, revealing the distribution of a factor's prognostic effect across the different cut‐points and measurement methods. Then, in Sections 3 and 4, we consider when each study potentially provides *multiple* prognostic results for each factor, relating to different cut‐points and/or methods of measurement. We show how multivariate meta‐analysis models can accommodate the correlation between such results 12 and allow summary meta‐analysis results to be produced for each cut‐point and method of measurement, thereby facilitating clinical interpretation. The multivariate approach handles missing results (e.g. for particular cut‐points or methods of measurement) in some studies and utilises correlation to gain more information, which is generally known to improve the statistical properties of meta‐analysis results compared with standard (univariate) approaches 14. We extend the general multivariate model to allow a functional relationship in the prognostic effect size over different values of the cut‐point, to improve model convergence and applicability. An application is made to real examples throughout, and Section 6 concludes with some discussion.

## 2 Meta‐analysis using one result per study

Let there be *i* = 1 to *k* studies available for meta‐analysis, and let each study provide just one prognostic effect estimate, *y*_*i*_, and its variance, $s_{i}^{2}$, for a particular continuous factor of interest when dichotomised at some cut‐point and measured using a chosen method of measurement. The *y*_*i*_ will typically be either a log hazard ratio or a log odds ratio estimate. When studies use different cut‐points and methods of measurement, a sensible option is to perform a separate meta‐analysis for each subset of studies that used the same measurement and cut‐point. However, in practice, we rarely see this approach, probably because most subsets contain only a few studies. It is more common to see researchers meta‐analyse all studies together and account for potential between‐study heterogeneity in prognostic effects using a random‐effects model:

(1) $$\begin{array}{cc} y_{i} & ∼N\left(\theta_{i},s_{i}^{2}\right)\\ \theta_{i} & ∼N\left(\beta ,\tau^{2}\right) \end{array}$$

In model (1), the $s_{i}^{2}$ is assumed known, which is a common assumption in the meta‐analysis field 15, and each study's true prognostic effect, *θ*_*i*_, is assumed normally distributed about a summary (mean) prognostic effect, *β*, with between‐study variance, *τ* ^2^. The model can be estimated using, for example, restricted maximum likelihood (REML) or methods of moments 16. The major problem with this approach is that the summary effect, *β*, is difficult to interpret clinically as it does not relate to a particular cut‐point or method of measurement. If one adopts this model, we argue that it is better to focus on the range of prognostic effects across studies by calculating a prediction interval for the potential prognostic effect of the factor in a new study 13, 17, 18 by

(2) $$\left[\hat{\beta }-t_{N-2}\sqrt{{\hat{\tau }}^{2}+\mathrm{Var}(\hat{\beta })},\hat{\beta }+t_{N-2}\sqrt{{\hat{\tau }}^{2}+\mathrm{Var}(\hat{\beta })}\;\right]$$

where Var( $\hat{\beta })$ is the variance of $\hat{\beta }$, and *t*_*N* − 2_ is the 100(1 − *α*/2) percentile of the *t*‐distribution with *N* − 2 degrees of freedom, with *α* usually chosen as 0.05 to give a 95% prediction interval. A *t*‐distribution, rather than a normal distribution, is used to help account for the uncertainty in ${\hat{\tau }}^{2}$ 13.

If the entire prediction interval does not include the value of no effect (e.g. a log odds ratio or log hazard ratio of 0), this suggests that the factor is likely to have prognostic value in new populations that use similar cut‐points and methods of measurement to those in the included studies. If the interval contains the value of no effect, this indicates the factor may not be prognostic in at least some situations, and the reasons for this could then be explored. For example, if the number of studies is sufficient (e.g. > 10), then the association of the cut‐point value (*x*_*i*_) and the prognostic effect can be examined in a meta‐regression by

(3) $$\begin{array}{cc} y_{i} & ∼N\left(\theta_{i},s_{i}^{2}\right)\\ \theta_{i} & ∼N\left(\alpha +\gamma_{1}x_{i},\tau^{2}\right) \end{array}$$

where *γ*_1_ gives the expected change in the summary prognostic effect for a 1‐unit increase in the cut‐point value. After the estimation of model (3), the summary prognostic effect estimate for a particular cut‐point is then obtained by $\hat{\alpha }+{\hat{\gamma }}_{1}x_{i}$. Similarly, covariates could be included for the method of measurement.

***Example: Ki‐67 as a prognostic factor in breast cancer patients***

**Figure 1:**
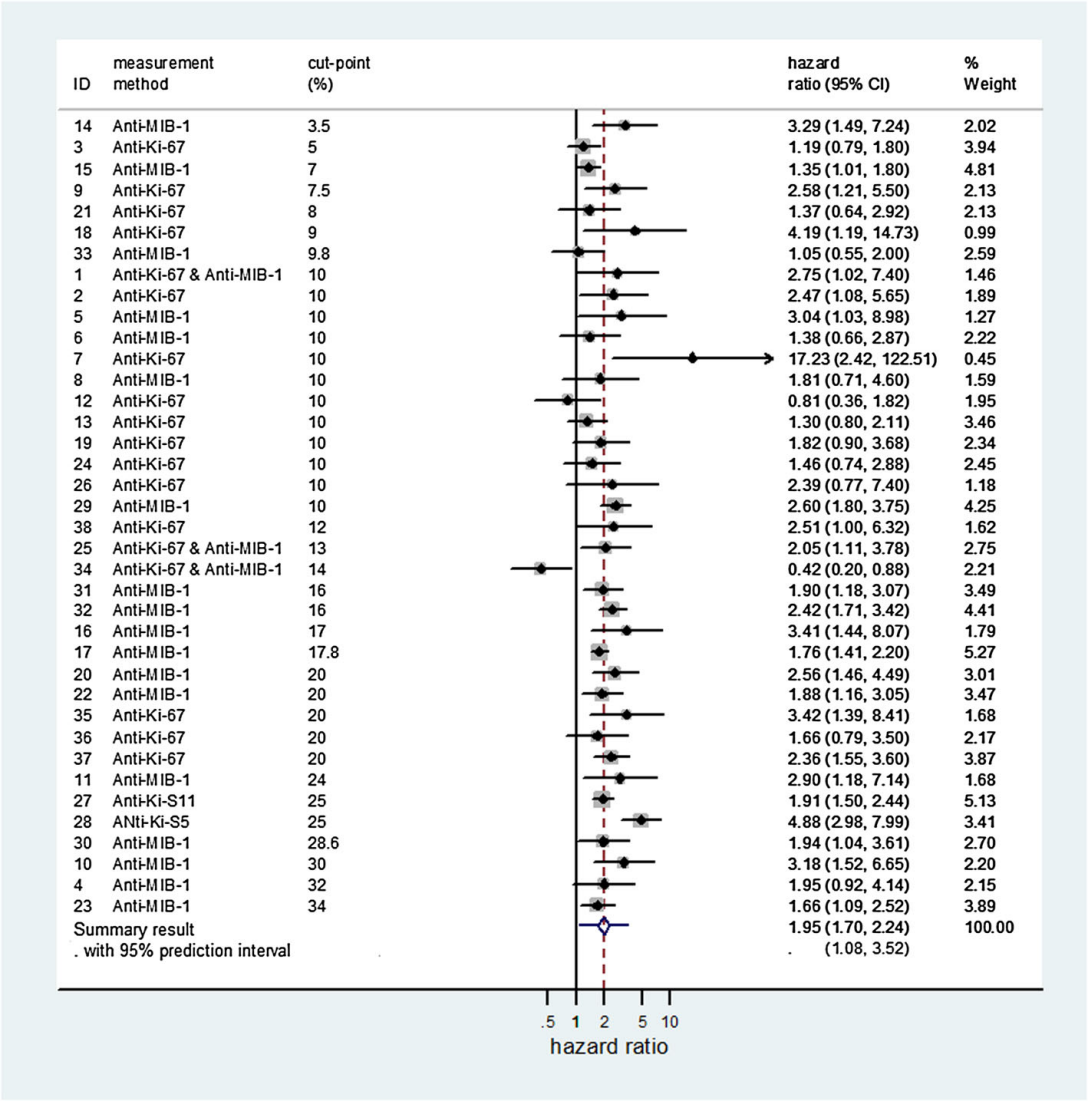
Forest plot of the study estimates and meta‐analysis results for the prognostic effect of Ki‐67 for overall survival in patients with breast cancer.

The Ki‐67 antigen is used to evaluate the proliferative activity of breast cancer, and is recorded on a continuous scale (as a % of tumour cells that are active). De Azambuja *et al.* 11 examine whether Ki‐67 is a prognostic factor for overall survival of patients with breast cancer. Across 35 studies identified, 38 unadjusted hazard ratios were obtained for independent groups of patients. The study cut‐points ranged from 3.5% to 34%, and five different methods were used to measure Ki‐67 activity (Figure 1). Applying model (1) produces a summary log hazard ratio of 0.67 (95% CI: 0.53 to 0.81), corresponding to a summary hazard ratio of 1.95 (95% CI: 1.70 to 2.24) (Figure 1). This reveals that – *on average* across all cut‐points, methods of measurement and other heterogeneous factors – Ki‐67 has prognostic value, with higher values of activity associated with a higher rate of mortality.

Unsurprisingly, there is a large heterogeneity in the meta‐analysis ( $I^{2}=52.3\%;\hat{\tau }=0.28)$. A 95% prediction interval for the prognostic effect of Ki‐67 in a new population which, using equation (2), is 1.08 to 3.52 (Figure 1). The interval is entirely above 1, suggesting that Ki‐67 has prognostic value across all the cut‐points and methods of measurement used in the 35 studies included in the meta‐analysis. Applying model (3) provided no evidence that either the cut‐point ( ${\hat{\gamma }}_{1}=$0.008, 95% CI: ‐0.012 to 0.028, p = 0.43), or the method of measurement (Anti‐Ki‐67 or Anti‐MIB‐1; p = 0.77) were associated with the prognostic effect of Ki‐67.

## 3 Meta‐analysis using multiple cut‐point results per study

Model (1) only uses one result per study, but in many studies multiple prognostic results will be available for a particular factor. Consider now that each study in the meta‐analysis uses the same method of measurement for a particular factor but may provide *multiple* prognostic effect estimates for a range of different cut‐points. To accommodate multiple estimates per study, we use a multivariate meta‐analysis model 12, 19 that accounts for *within‐study correlation* of the multiple prognostic effect estimates 20 (caused by the same patients contributing to each cut‐point estimate) and any *between‐study correlation* in the true effects at each cut‐point. The model is now detailed in full.

### 3.1 A general model for multivariate meta‐analysis of studies with multiple cut‐points

Without loss of generalisability, assume that there is a prognostic effect estimate *y*_*ij*_ and its variance, $s_{\mathrm{ij}}^{2}$, for each cut‐point of up to *j*=*1* to *T* different cut‐points per study (*i* = 1to*k*), and let these cut‐points be ordered in an increasing value. Further, assume that the within‐study covariance between each pair of cut‐points (e.g. *c* *o* *v*_*i*(1,*T*)_ is the within‐study covariance between *y*_*i*1_ and *y*_*iT*_; the estimates for cut‐points 1 and *T*, respectively) is known. Section 3.3 discusses how to obtain the within‐study covariances, or how to proceed if they are not available. If all studies report all cut‐points, the general multivariate normal random‐effects meta‐analysis model assumes that 12

(4) $$\begin{array}{c} \left(\begin{array}{c} y_{i1}\\ y_{i2}\\ \vdots \\ y_{\mathrm{iT}} \end{array}\right)∼N\left(\left(\begin{array}{c} \theta_{i1}\\ \theta_{i2}\\ \vdots \\ \theta_{\mathrm{iT}} \end{array}\right),\left(\begin{array}{cccc} s_{i1}^{2}\\ \mathrm{co}v_{i(1,2)} & s_{i2}^{2}\\ \vdots & \vdots & \ddots \\ \mathrm{co}v_{i(1,T)} & \mathrm{co}v_{i(2,T)} & \cdots & s_{\mathrm{iT}}^{2} \end{array}\right)\right) \end{array}$$

where

$$\begin{array}{c} \left(\begin{array}{c} \theta_{i1}\\ \theta_{i2}\\ \vdots \\ \theta_{\mathrm{iT}} \end{array}\right)∼N\left(\left(\begin{array}{c} \beta_{1}\\ \beta_{2}\\ \vdots \\ \beta_{T} \end{array}\right),\Omega \right) \end{array}$$

In model (4), **Ω** is the between‐study variance–covariance matrix for the true log hazard ratios and, if unstructured, is a *T* by *T* matrix containing *T* between‐study variances (one for each cut‐point, e.g. $\tau_{1}^{2}$ for cut‐point 1) in the diagonal and (2*T‐1)*between‐study covariances in the off‐diagonals (e.g. one for each pair of cut‐points, e.g. *τ*_1,*T*_ for cut‐points 1 and *T*) :

$$\begin{array}{c} \Omega =\left(\begin{array}{cccc} \tau_{1}^{2}\\ \tau_{1,2} & \tau_{2}^{2}\\ \vdots & \vdots & \ddots \\ \tau_{1,T} & \tau_{2,T} & \cdots & \tau_{T}^{2} \end{array}\right) \end{array}$$

The study estimates (*y*_*ij*_), their variances $\left(s_{\mathrm{ij}}^{2}\right)$, and covariances (e.g. *c* *o* *v*_*i*(1,*T*)_) are required to fit model (4). As for model (1), the within‐study variances and, additionally here, the within‐study covariances are assumed known.

Crucially, the model can accommodate missing results for some cut‐points in a study, assuming they are missing at random 21, just as described elsewhere for missing outcomes in a multivariate meta‐analysis of multiple outcomes 12, 14. In other words, the probability that a particular *y*_*ij*_ is missing for a cut‐point depends solely on the observed *y*_*ij*_ for other cut‐points and not on the actual value of the missing *y*_*ij*_ itself. Interestingly, even when data are not missing at random, this multivariate meta‐analysis model has been shown to obtain summary estimates with improved statistical properties compared with univariate meta‐analysis 12, 20, 22. For example, if some cut‐points are selectively missing because of their actual value of *y*_*ij*_ (such as, always available if the corresponding odds ratio is statistically significant, but often unavailable if non‐significant), then the missing data are missing not at random. The multivariate results are less biased than univariate results in this situation; although, the bias is not removed in full 22.

The model can be fitted using, for example, methods of moments 23, 24 or REML, using software such as SAS Proc MIXED 25 or the ‘mvmeta’ module in STATA 26. The ${\hat{\beta }}_{j}$ terms give the summary (mean) prognostic effect (e.g. log hazard ratio or log odds ratio) at cut‐point *j*.

### 3.2 Multivariate meta‐analysis assuming a functional relationship between summary prognostic effect and cut‐point

Model (4) is best suited to situations involving a small number of cut‐points across studies (e.g. 2 or 3), as otherwise, the number of parameters in the model is potentially large: one has to estimate *T* summary means, *T* between‐study variances and (*T* ^2^−*T*)/2 between‐study covariances (correlations). One could impose a structure to **Ω** to reduce the number of parameters to be estimated. For example, one could assume a common between‐study variance at each cut‐point and the same between‐study correlation for all pairs of cut‐point. This is potentially over‐simplistic, as the between‐correlation is likely to be higher for two neighbouring cut‐points than for two cut‐points far apart. Adopting an auto‐regressive structure for **Ω** may help address this, as done in linear mixed effects models with repeated measurements ordered over time.

Alternatively, one could assume a particular functional form for the relationship between the true prognostic effect and the cut‐point value. A similar idea has been proposed in meta‐analysis of test accuracy studies reporting multiple thresholds 27, 28 and is closely related to meta‐analysis of longitudinal data 29. For example, a linear relationship could be assumed such that a 1‐unit increase in cut‐point value, *x*_*j*_, leads to a constant change of *γ* in the prognostic effect

$$\begin{array}{c} \left(\begin{array}{c} y_{i1}\\ y_{i2}\\ \vdots \\ y_{iT} \end{array}\right)∼N\left(\left(\begin{array}{c} \alpha_{i}+\gamma x_{1}\\ \alpha_{i}+\gamma x_{2}\\ \vdots \\ \alpha_{i}+\gamma x_{T} \end{array}\right),\left(\begin{array}{cccc} s_{i1}^{2}\\ \mathrm{co}v_{i(1,2)} & s_{i2}^{2}\\ \vdots & \vdots & \ddots \\ \mathrm{co}v_{i(1,T)} & \mathrm{co}v_{i(2,T)} & \cdots & s_{\mathrm{iT}}^{2} \end{array}\right)\right) \end{array}$$

(5) $$\alpha_{i}∼N\left(\alpha ,\tau_{\alpha }^{2}\right)$$

where *x*_*j*_ is the *j* ^th^ cut‐point value (where *j* = 1 to *T*, and *T* is the total number of different cut‐points considered across studies, ordered in increasing value). In model (5), *α* is the average intercept (the summary prognostic effect when the cut‐point = 0) and $\tau_{\alpha }^{2}$ is the between‐study variance in the intercept. The slope, *γ*, gives the summary change in log hazard ratio (odds ratio) for a 1‐unit increase in the cut‐point value. Extensions to model (5) may specify random slopes, but this may not be practical if some studies only provide results for one or two cut‐points.

Following estimation of model (5), for example using REML, summary estimates for the log hazard ratio (odds ratio) at a particular cut‐point, *t*, can be obtained by $\hat{\alpha }+\hat{\gamma }x_{j}$. Confidence and prediction intervals can be obtained as before. SAS code to fit model (5) is given in Supplementary Material 1.

***Nonlinear extensions***

Model (5) has substantially less parameters to estimate than model (4), especially when the number of cut‐points is large. However, this computational advantage comes at the expense of assuming a particular functional relationship between prognostic effect size and cut‐point value. The linear relationship specified in model (5) may not be appropriate, and rather a nonlinear trend may be preferable, for example using restricted cubic splines or fractional polynomials 30. Model fit statistics can help identify the best fit, but when the number of studies and cut‐points are small, the power to detect the true relationship is likely to be low.

### 3.3 Obtaining within‐study correlations

Models (4) and (5) require the within‐study covariances (e.g. *c* *o* *v*_*i*(1,*T*)_), or equivalently the within‐study correlations (e.g. *ρ*_*Wi*(1,*T*)_=*s*_*i*1_ *s*_*iT*_ *c* *o* *v*_*i*(1,*T*)_) between pairs of prognostic effect estimates. These are unlikely to be available from publications 20, 31. If individual participant data (IPD) are available, non‐parametric bootstrapping is a general method that can be used to obtain them as described elsewhere 32, 33. Where the within‐study correlation between two unadjusted odds ratios are of interest, the necessary IPD can be reconstructed if the two by two tables at each cut‐point are available (giving the number of patients above and below the cut‐point, and the number of events in each group), from which bootstrapping can proceed. If a study's IPD (or subsequent bootstrap samples) produce a two by two table with a zero cell, odds ratios can be calculated by adding a continuity correction to the cells: we suggest the approach of Sweeting *et al.* 34, who add 1/(sample size of the opposite group) to each cell.

For situations where the effect estimates are adjusted odds ratios, unadjusted hazard ratios, or adjusted hazard ratios, it is unlikely that IPD can be recreated from published information to allow within‐study correlations (covariances) to be derived via bootstrapping. One could then impute plausible values for the missing within‐study correlation. For example, if correlations *are* available for unadjusted but not adjusted results, then one might assume the former is a close approximation for the latter 35. Alternatively, one could seek clinical opinion, identify within‐study correlations from related studies or perform sensitivity analyses across a range of values 20. In particular, if some studies do provide IPD, then the within‐study correlations can be derived in these studies and assumed to be the same in non‐IPD studies. A Bayesian approach would also allow a prior distribution to be specified for the missing within‐study correlations 33, 36, 37. Hedges *et al.* 38 propose a robust variance estimation approach for meta‐regression with correlated effect sizes but unknown correlations, which they suggest provides accurate results when there are at least 20 studies. Also, Riley *et al.* 39 propose an alternative ‘overall correlation’ multivariate model that does not require the within‐study covariances to be specified, as it includes just one overall correlation term (an amalgamation of the within and between‐study correlations), but performs well in terms of estimation of the *β*_*j*_s, although it may fail to converge if the between‐study heterogeneity is small relative to the within‐study variances.

### 3.4 Example: Apgar score as a prognostic factor of neonatal outcomes

The Apgar score is measured in babies immediately after birth 40. It ranges from 0 to 10, with lower values considered to be strongly associated with a higher risk of neonatal mortality, morbidity and childhood cerebral palsy. Malin *et al*. 41 performed a systematic review of the prognostic ability of the Apgar score in babies who weigh less than 2500g in relation to neonatal mortality and identified differences in the cut‐points used in each study. Here, we use their data to illustrate the multivariate meta‐analysis methods described previously.

#### 3.4.1 Consideration of two cut‐points

First, consider those 10 studies reporting prognostic results for the two most frequently used cut‐points, 3 and 6, where values less than or equal to the cut‐point are defined as ‘poor’. Five studies presented prognostic results for both cut‐points, four studies considered just cut‐point 3 and one study considered just cut‐point 6. The unadjusted odds ratio estimates for each cut‐point are shown in Table 1 for each study. In those five studies that provide results for both cut‐points, the two estimates have moderately high within‐study correlations around +0.5 (calculated using bootstrapping). Multivariate model (4) uses these correlations to gain more information (‘borrow strength’ 12), thereby limiting the missing results for some cut‐points in some studies, especially for cut‐point 6.

**Table 1:** Prognostic effect estimate for the Apgar score at each available cut‐point in each study, where the outcome is neonatal mortality.

	Cut‐point 3		Cut‐point 6			
	Log odds	SE of log	Log odds	SE of log	Within‐study	Within‐study
Study ID	ratio	odds ratio	ratio	odds ratio	covariance	correlation
1	2.599	0.136	2.383	0.153	0.012	0.589
2	1.980	0.197	2.210	0.301	0.027	0.456
3	2.920	0.194	2.606	0.234	0.026	0.580
4	3.265	0.149	2.997	0.177	0.014	0.529
5	2.256	0.294	1.939	0.239	0.043	0.613
6	1.609	0.305	—	—	—	—
7	1.314	0.237	—	—	—	—
8	2.311	0.421	—	—	—	—
9	0.806	0.317	—	—	—	—
10	—	—	2.386	0.447	—	—

Odds ratios are defined as the odds ratios of death for those with an Apgar score ≤ cut‐point value, divided by the odds of death for those with an Apgar score > cut‐point value. SE, standard error.

Table 2 compares the results of a separate univariate meta‐analysis (model (1)) for each cut‐point with those from multivariate meta‐analysis model (4). The summary odds ratio for cut‐point 3 is very similar for all analyses, between 8.5 and 8.7. However, for cut‐point 6 the summary odds ratio is substantially lower in the multivariate analyses. The univariate analysis gives a summary odds ratio of 11.56 (95% CI: 8.35 to 15.99), but multivariate model (4) gives a summary odds ratio of 7.93 (95% CI: 5.17 to 12.16). The between‐study correlation is poorly estimated at +1, a common occurrence in multivariate meta‐analysis 42. The ‘overall correlation’ model of Riley *et al.* 39, which does not require within‐study correlations and avoids estimating the between‐study correlation, estimates an overall correlation of +0.948 and produces a summary odds ratio of 8.25, again substantially lower than the univariate solution. Indeed, in contrast to univariate results, the multivariate results suggest cut‐points 3 and 6 have similar prognostic effects. The multivariate also gives noticeably narrower confidence intervals (Table 2). These findings are due to the multivariate meta‐analysis, under the missing at random assumption, reducing the impact of missing cut‐point results by borrowing strength from other correlated cut‐point results that are available.

**Table 2:** Meta‐analysis results for the prognostic effect of the Apgar score at each cut‐point, where the outcome is neonatal mortality.

	Cut‐point 3			Cut‐point6				
Analysis method	Summary log odds ratio (SE)	Summary odds ratio [95% CI]	${\hat{\tau }}_{1}$	Summary log odds ratio (SE)	Summary odds ratio [95% CI]	${\hat{\tau }}_{2}$	Between‐study correlation $={\hat{\tau }}_{12}/{\hat{\tau }}_{1}{\hat{\tau }}_{2}$	Overall correlation*
Univariate model (1)	2.14 (0.264)	8.50 [5.06, 14.27]	0.75	2.45 (0.166)	11.56 [8.35, 15.99]	0.319	—	—
Multivariate model (4)	2.16 (0.246)	8.69 [5.37, 14.07]	0.72	2.07 (0.218)	7.93 [5.17, 12.16]	0.560	1	—
Alternative Multivariate model*	2.14 (0.247)	8.52 [5.25, 13.81]	0.72	2.11 (0.205)	8.25 [5.52, 12.34]	0.502	—	0.95

^*^Using the alternative model of Riley *et al.* 39, which does not require within‐study correlations

The $\hat{\tau }$ relate to the log odds ratio scale

Odds ratios are defined as the odds of death for those with an Apgar score ≤ cut‐point value, divided by the odds of death for those with an Apgar score > cut‐point value. SE, standard error.

Using the model (4) estimates, 95% prediction intervals are 1.50 to 50.29, and 2.01 to 31.25 for cut‐points 3 and 6, respectively. These suggest that the prognostic effect of the Apgar score will vary greatly in magnitude across populations even when the same cut‐point is used, because of unexplained heterogeneous factors. However, the effects are consistently in the same direction, such that lower values of the Apgar score indicate a higher risk of neonatal mortality.

#### 3.4.2 Consideration of multiple cut‐points and a functional relationship

In the previous example, just two cut‐points were considered for illustration. However, there were actually 10 different cut‐points considered by a total of 11 studies identified by the review (Supplementary Material 2). One study examined all 10 cut‐points, but the other studies just examined one or two cut‐points. Only cut‐points 3 and 6 were evaluated by more than two studies. As two by two tables were available for all reported cut‐points, IPD were recreated and bootstrapping used to obtain the within‐study covariance estimates (available on request).

Model (5) was applied to the dataset using REML, and thus a linear relationship estimated between the prognostic effect size and the cut‐point value. The estimate of the average intercept ( $\hat{\alpha })$ was 2.43 (95% CI: 1.95 to 2.91; *p* < 0.001), which suggests that the odds of death for babies with an Apgar score of zero are 11.36 (= exp(2.43)) times those for babies with an Apgar value greater than zero. The estimate of the slope ( $\hat{\gamma })$ was −0.068 (95% CI: −0.11 to −0.025; *p* = 0.002), indicating that the log odds ratio (comparing those below with those above the cut‐point) decreases as the cut‐point increases. There was substantial between‐study heterogeneity in the intercept, with ${\hat{\tau }}_{\alpha }=0.67$, a similar value to that observed for cut‐points 3 and 6 (the most commonly reported cut‐points) in the previous multivariate analyses of Section 3.4.1 (Table 2). Allowing for additional heterogeneity in the slope made no difference as this was estimated as zero.

**Figure 2:**
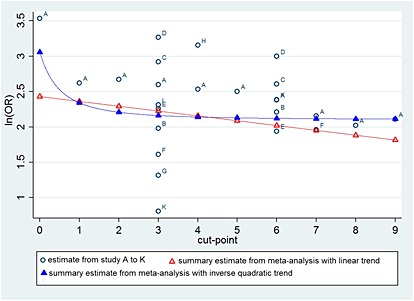
Comparison of the individual study estimates and the summary meta‐analysis results obtained from model (5), assuming either a linear trend or an inverse quadratic trend, for the prognostic effect of the Apgar score at each cut‐point, in relation to neonatal mortality. Confidence intervals around each point are not shown for cosmetic reasons, but are provided for the summary estimates in Table 3. Odds ratios are defined as the odds of death for those with an Apgar score ≤ cut‐point value, divided by the odds of death for those with an Apgar score > cut‐point value.

The model estimates allow a summary meta‐analysis result for each cut‐point, and these are shown in Table 3 and graphically in Figure 2. However, the linear relationship seems visually inappropriate, as the observed odds ratio estimates at a cut‐point of 0 appears to be substantially larger than those for other cut‐points. Model (5) was thus refitted using fractional polynomials and a selection procedure 30, 43, which marginally indicated that the cut‐point variable should be included as an inverse quadratic term (1/(cut‐point)^2^), rather than linear (AIC of linear = 1353.3, AIC of inverse quadratic = 1352.9). This nonlinear function provides noticeably higher summary odds ratios at the first and last cut‐point values than the linear function (Table 3 and Figure 2). The shape of the relationship is predominately based on the one study (labelled ‘A’ in Figure 2) that reported all cut‐points, which is actually the original Apgar study 40. However, compared with this single study, the summary curve provides odds ratios closer to 1 because it accounts also for the other studies (labelled B to K in Figure 2), which are generally giving estimates closer to 1 than study A. This is especially driven by cut‐point 3, the most commonly reported cut‐point, whose overall mean across all studies is much lower than its single estimate from study A. In summary, the shape of the relationship across cut‐points is driven by study A; however, the location is driven by cut‐point 3.

**Table 3:** Model (5) meta‐analysis results for the prognostic effect of the Apgar score at each cut‐point, where the outcome is neonatal mortality.

	*Model* (3) *with linear trend*		*Model* (3) *with inverse quadratic trend*		
Cut‐point	Summary odds ratio	95% CI	Summary odds ratio	95% CI	95% prediction interval
0	11.36	7.06 to 18.30	21.30	8.07 to 56.17	3.33 to 136.40
1	10.61	6.68 to 16.86	10.40	6.50 to 16.63	2.15 to 50.41
2	9.91	6.30 to 15.59	9.11	5.88 to 14.10	1.90 to 43.60
3	9.26	5.92 to 14.47	8.69	5.63 to 13.42	1.82 to 41.57
4	8.65	5.54 to 13.49	8.51	5.51 to 13.13	1.78 to 40.69
5	8.07	5.17 to 12.62	8.41	5.44 to 12.99	1.76 to 40.23
6	7.54	4.80 to 11.86	8.35	5.40 to 12.90	1.75 to 39.95
7	7.04	4.44 to 11.18	8.31	5.38 to 12.85	1.74 to 39.77
8	6.58	4.09 to 10.58	8.29	5.36 to 12.81	1.73 to 39.65
9	6.14	3.76 to 10.05	8.27	5.35 to 12.78	1.73 to 39.57

The summary curve and estimated model parameters can be used to estimate a summary odds ratio at each cut‐point (Table 3). Compared with analysing each cut‐point separately, the summary estimates are closer to 1 for most cut‐points than the observed estimates might suggest (in other words, the fitted relationship may appear visually a poor fit in Figure 2). However, as described in Section 3.4.1, this is due to other cut‐points utilising the correlated information from the available cut‐points 3 and 6 results, which are reported more often.

Interestingly, there is very little difference in the summary odds ratio when using cut‐points from 3 to 9, but bigger changes occur when using cut‐points below 3. The largest summary odds ratio is seen for a cut‐point of zero. Given the large heterogeneity, it is important to present 95% prediction intervals for the odds ratio at each cut‐point (Table 3). All intervals are above 1 and incredibly wide; for example, the 95% prediction intervals at cut‐points 3 and 6 are 1.82 to 42 and 1.75 to 40, respectively.

## 4 Meta‐analysis with results for multiple measurement methods per study

The models in Section 3 can be applied or extended to deal with multiple results for different methods of measurement per study. A multivariate approach is appropriate because the different methods of measurement are often correlated at the patient‐level, which induces correlation amongst the prognostic effects for the measurements. We now briefly outline the model framework, followed by an example.

### 4.1 Multiple methods of measurement per study, but consistent specification of the prognostic factor

Assume that, for each method of measurement considered in the studies for meta‐analysis, there is a consistent specification of the candidate prognostic factor; that is, the same cut‐point is used in all studies, or it is always modelled as a linear trend. In this situation, multiple effect estimates arise only because of the multiple methods of measurement, which could be written as *y*_*ij*_ where now *j* = 1 to *M* (rather than *j* = 1 to *T*, as written earlier). Thus, model (4) is now applicable again, with ${\hat{\beta }}_{j}$ now giving the summary prognostic effect for the *j* ^th^ method of measurement.

As before, model (4) requires within‐study correlations (covariances) to be known. Given IPD bootstrapping can still be used to obtain them. If IPD are not available, then studies that report multiple measurement results often provide the patient‐level correlation between the methods of measurement. This could be used to approximate the within‐study correlation of the prognostic effect estimates from the methods. For example, Wei and Higgins provide a formula for the within‐study covariance for two unadjusted log odds ratio estimates 31. This might also be used to approximate the within‐study correlation between two adjusted log odds ratio estimates. In other situations (e.g. when dealing with unadjusted or adjusted hazard ratios), one could use the patient‐level correlation itself as approximation for the within‐study correlation. The alternative ‘overall correlation’ multivariate model 39 can again be fitted without within‐study correlations.

### 4.2 Multiple methods of measurement and cut‐points

If there are *both* multiple methods of measurement and multiple cut‐points, then the multivariate models in Section 3 can be extended, with each method of measurement in each study providing a set of *y*_*ij*_s for the meta‐analysis, thereby enabling a summary mean prognostic effect to be obtained for each method at each cut‐point. To keep the number of parameters to a minimum, this is best achieved by extending model (5), and thereby assuming a functional relationship across cut‐points for each method of measurement. For example, if there are two methods of measurement, then model (5) can be extended to include a separate intercept and slope for each method. A different between‐study variance could also be assumed for each intercept, and then a between‐study correlation may also be needed.

### 4.3 Example: Microvessel density as a prognostic factor in non‐small‐cell carcinoma

Trivella *et al.* 44 assess whether microvessel density counts (a measure of angiogenesis) are a prognostic factor of mortality in patients with non‐small‐cell lung carcinoma. IPD were obtained from 16 studies, and the hazard ratio for a 1‐unit change in microvessel counts was calculated in each study, after adjusting for age and tumour size. Thus, the prognostic effect is specified consistently across studies. However, two methods of measurement were used by the studies: the Chalkley method and the ‘counting all microvessels’ method. Three studies used both methods of measurement and so provided two adjusted hazard ratios, one for each method. In the other 13 studies, only one method of measurement was used, and so only one adjusted hazard ratio was available for either the Chalkley method (three studies) or the all vessels method (10 studies). Multivariate meta‐analysis model (4) allows the joint analysis of both methods of measurement to account for their correlation and thereby reduce this missing data problem. Log hazard ratios estimates and their standard errors are shown for each study in Table 4. For those three studies with results for both methods of measurements, within‐study correlations were not reported by Trivella *et al.,* but the patient‐level correlations were given as 0.55, 0.74 and 0.27, respectively. Here, we take these as an approximation for the missing within‐study correlations (Table 3), but recognise with the original IPD, one could use bootstrapping to obtain them.

**Table 4:** Prognostic effect estimate for a 1‐unit change in microvessel density, for each method of measurement in each study, where the outcome is mortality.

	*Chalkley method*		*All vessels method*			
Study ID	Log hazard ratio	SE of log hazard ratio	Log hazard ratio	SE of log hazard ratio	Within‐study covariance*	Patient‐level correlation
1	0.122	0.087				
3	0.039	0.06				
4			−0.02	0.09		
5			0.058	0.063		
6	0.104	0.065	0.02	0.091	0.0033	0.55
7	0.039	0.038				
8			0.239	0.039		
9			−0.211	0.221		
10			0.03	0.061		
11			−0.01	0.02		
12			0.307	0.252		
13	0.02	0.066	0	0.025	0.0012	0.74
14			−0.693	0.758		
15			−0.174	0.142		
16			−0.02	0.025		
17	0.03	0.047	0.049	0.037	0.00048	0.27

^*^Assuming within‐study correlation is equal to the patient‐level correlation.

NB data derived from the reported hazard ratios and CIs in Figures 1 and 2 of Trivella *et al*. 44 SE, standard error.

Trivella *et al.* 44 performed a separate univariate meta‐analysis (model (1)) for each method of measurement. They concluded that microvessel density was not prognostic when using the all vessels method, and there was only weak evidence that it was prognostic when using the Chalkley method. Importantly, when accounting for within and between‐study correlations, the multivariate meta‐analysis model (4) reaches the same conclusion (Table 5). Summary hazard ratio estimates are very similar, although confidence intervals are slightly narrower. Even when assuming large within‐study correlations of +0.9, summary estimates and heterogeneity estimates barely change. Thus, this additional analysis suggests that the original meta‐analysis results of Trivella *et al.* are robust after accounting for the correlation in the results for the two measurement methods. Finally, we note that the ‘overall correlation’ model of Riley *et al.* 39 does not converge for this example, most likely due to the miniscule heterogeneity for the Chalkley method causing estimation difficulties for the overall correlation.

**Table 5:** Meta‐analysis results for the prognostic effect of a 1‐unit increase in microvessel density for each method of measurement, where the outcome is mortality.

	*Chalkley method*		*All vessels method*		
Analysis method	Summary hazard ratio (95% CI)	${\hat{\tau }}_{1}$	Summary hazard ratio (95% CI)	${\hat{\tau }}_{2}$	Between‐study correlation $={\hat{\tau }}_{12}/{\hat{\tau }}_{1}{\hat{\tau }}_{2}$
Univariate model (1)	1.049 [1.004, 1.096]	<1 x 10^−13^	1.032 [0.973, 1.093]	0.077	—
Multivariate model (4)	1.051 [1.007, 1.097]	0.0025	1.030 [0.972, 1.091]	0.077	1

## 5 Discussion

Empirical evaluations, across a wide‐range of disease fields, have shown that meta‐analysis of prognostic factor studies is often limited by heterogeneity and missing results in primary studies 1, 45. In this article, we have suggested meta‐analysis approaches that allow more clinically useful results about prognostic factors in the presence of heterogeneity. In particular, we have focused on multivariate meta‐analysis methods to examine prognostic value at particular cut‐points and for specific methods of measurement. As they utilise more information, multivariate meta‐analysis methods are being used to synthesise multiple treatment comparisons 46 and multiple outcomes 47. Here, under a missing at random assumption, the utilisation of correlation reduces the impact of missing results for particular cut‐points and methods of measurement in some studies 14, 22. Even when data are not missing at random, the multivariate meta‐analysis model is likely to obtain more appropriate inferences than current univariate approaches, as the correlation reduces (although does not entirely remove) the impact of selectively missing results 12, 20, 22. In our examples, the multivariate approach revealed important insight about the prognostic value of the Apgar score and microvessel density. In particular, in the Apgar example, the multivariate approach produced substantially lower summary estimates at some cut‐points than previously thought. For example, in Table 2 the univariate meta‐analysis suggests a cut‐point of ≤ 6 gives a larger prognostic effect, whereas the multivariate meta‐analysis suggests it is slightly higher for cut‐point 3. The latter is clinically more intuitive, as lower values are considered to put babies at a higher risk, and so lower cut‐points are expected to lead to higher odds ratios.

**Usefulness of prognostic factor effects based on a cut‐point**

There are a number of clinical applications where a cut‐point may be useful for implementing the prognostic factor. For example, in complex health economic models or disease outcome simulation models 48, for parsimony, a population may be divided into two groups defined by a prognostic factor with a cut‐point. In randomised trials that incorporate prognostic factors in the randomisation process (e.g. within minimisation or stratification), it may be more convenient to consider dichotomised factors. The actual analysis of trials (or indeed observational studies) may specify, *a priori*, a set of prognostic factors (confounders) to be adjusted for 49; their inclusion may be based on evidence in previous studies, for which prognostic value at cut‐points may only be known. In prognostic models for clinical risk prediction, prognostic factors within the risk equation are sometimes categorised to ease implementation by clinicians and health professionals 50. In clinical decision making, treatment decisions may be informed by prognostic factor values above a cut‐point. For example, the use of drug‐eluting stents for the treatment of coronary artery disease was restricted by The National Institute for Health and Clinical Excellence to patients with coronary artery lesions longer than 15mm 51, a prognostic factor for the probability of restenosis, as patients with such lesions had a worse prognosis and thus were considered to have a greater potential to gain from treatment. In such examples, knowledge of the absolute risk in each of the groups defined by the prognostic factor is clearly important, not just the relative risks of the two groups.

**Linear and nonlinear prognostic effects**

Although cut‐point specific results can be useful, it is well known that dichotomising continuous factors loses statistical power to detect their true prognostic effect 52. Therefore, the prognostic ability of a factor is better examined on its continuous, rather than dichotomised, scale and its linear or non‐linear relationship with outcome risk examined 53. Sauerbrei and Royston 54, and Gasparrini *et al.* 55 extensively discuss this approach when IPD are available for meta‐analysis. In the microvessel density example, we *were* able to examine the prognostic effect of a 1‐unit increase in the factor, for each of two different methods of measurement, as the original meta‐analysts used the IPD to analyse the factor on its continuous scale in each study. Analysing prognostic factors on their continuous scale is especially important in risk prediction research, where prognostic models are required to predict absolute outcome risk for individuals. Maintaining a continuous scale improves the range of possible predictions from the model, and is more likely to lead to a generalisable model than when including factors dichotomised. This is a major reason why IPD is increasingly sought when developing such models from multiple studies 56.

However, without IPD, meta‐analysts will predominately have to use reported prognostic results, which are most typically given for two groups defined by a cut‐point. In this situation, if researchers still want to examine the effect of a 1‐unit increase in a prognostic factor on outcome risk, then meta‐analysis approaches for examining dose‐response relationship are potentially applicable, as proposed by Greenland and Longnecker 35, and extended by others, for example 57, 58, 59. To apply these methods, some additional knowledge of the factor's underlying distribution is usually needed, as a particular factor value needs to be assigned to all patients within each group defined by a cut‐point (e.g. take the mid‐point or median) so that the trend across groups can be estimated in each study, to then be pooled in a meta‐analysis. The choice of such value can impact upon the results 57.

**Modelling issues and solutions**

Researchers wishing to implement our multivariate approaches should recognise potential modelling issues. We showed how to model the functional relationship between prognostic effect size and cut‐point value, and this will often be the most useful approach as it substantially reduces the number of parameters to estimate. Another practical issue is the derivation of within‐study correlations. These are most easily derived using bootstrapping when IPD are available 60. For unadjusted effects of dichotomous prognostic factors for a binary outcome, the IPD can be reconstructed from the published two by two table, as discussed in Section 3.3. However, in other situations, the IPD must be obtained directly from the original study authors, which might not be possible. When the IPD are not available, we showed how the multivariate approach might be implemented by using the patient‐level correlations 31 or a reparameterised multivariate model that does not require within‐study correlations 39. If the number of studies is very large (>20), then the robust variance approach of Hedges *et al*. 38 would allow the functional relationships to be modelled even when the within‐study correlations are unknown. Even obtaining IPD from just a single study can help, as within‐study correlations might be assumed exchangeable to other studies 33. Nevertheless, further methodological research on how to derive within‐study correlations from published data is needed, as this would undoubtedly improve the uptake of the multivariate models proposed here.

**Limitations of our work**

Our multivariate models with a functional relationship should not be extrapolated outside the range of cut‐points available in the studies for meta‐analysis. Furthermore, the multivariate models are unlikely to be reliable where most studies just report one cut‐point, especially if that cut‐point was selected on the basis of optimising the *p*‐value 61. In other words, the multivariate models are likely to perform best when at least one study has a large number of the cut‐points of interest, so that the relationship of prognostic effect sizes across cut‐points is based primarily on within‐study information, rather than between‐study information that is more prone to ecological bias and study‐level confounding 62.

We note also that, in some situations, there may be a known transformation that maps values from one method of measurement to another method of measurement. Clearly, if one can reliably transform findings from one scale to the other, then this is preferable to our approach and can be used to obtain missing method of measurement in studies that only report a subset of those of interest. However, in many situations, the relationship between competing methods is not known with high precision, or only applicable if the IPD are available. In this situation, our suggested joint multivariate meta‐analysis of all methods is then useful to account for their correlation.

Our models dealing with multiple cut‐points provide prognostic effect sizes such as odds ratios or hazard ratios. However, they do not provide absolute risks for those above and below a particular cut‐point. To derive absolute risks following our model, additional information would be required such as the absolute risk in at least one of the groups defined by a cut‐point and the distribution of the factors values, such that the proportion of the population that fall in between each cut‐point can be derived. However, absolute risks are typically more important from a prognostic model (which contains multiple prognostic factors in combination) 63, whereas here, the focus is on whether a particular factor has prognostic value 1. Furthermore, absolute risks tend to only be applicable to specific populations, whereas relative effects (such as odds ratios) are often more reasonably transportable between populations.

**Is meta‐analysis sensible?**

Other researchers have dealt with multiple cut‐points and methods of measurement by transforming to a standardised scale 64, 65, under various assumptions. This is often at the expense of clinical interpretability, as the scale then does not translate to a real metric for use. Our approaches rather produce results that translate directly to specific cut‐points and methods of measurement. However, the methods do not solve all the problems, as other heterogeneous factors remain unaddressed by our work, such as different adjustment factors 32 and stages of disease across studies. In some situations, heterogeneity may be considered too large to warrant meta‐analysis, and researchers should always exercise epidemiological and clinical judgement before pooling. If meta‐analysis is deemed sensible, then the pooled result may still be difficult to interpret when there is heterogeneity, and so in Section 2, we proposed that it is better to focus on the range of prognostic effects across studies by calculating a prediction interval. IPD can help reduce heterogeneity in prognostic factor studies, as seen in the microvessel density example where the heterogeneity was zero for both methods of measurement, following extensive data cleaning and standardisation of statistical analysis method and adjustment factors in each study 44. However, IPD often does not solve all the problems for meta‐analysis of prognostic factor studies 66, and in particular, publication bias related issues are a strong concern for this field 6, 7. Indeed, multivariate meta‐analysis may still be important with IPD to reduce the impact of (selectively) missing results 22.

## 6 Conclusion

In conclusion, we have proposed approaches for handling different cut‐points and methods of measurement in a meta‐analysis of prognostic factor studies. These are especially important when synthesising published prognostic results but are also potentially useful when IPD are available for some or all studies. Many issues remain in this field, and ultimately, a move toward prospectively planned pooled analyses would be preferred 67.

## Supporting information

Supporting info item
