# Supplementary material for: Multivariate meta‐analysis of prognostic factor studies with multiple cut‐points and/or methods of measurement
Source: Stat Med. 2015 Apr 29;34(17):2481–96. doi: 10.1002/sim.6493 (PMC4973834; doi:10.1002/sim.6493)
Supplement: Supplementary file 1 — Supporting info item [file SIM-34-2481-s001.docx]

**Supplementary Material S1: SAS Proc Mixed code to fit model (5) to the Apgar meta-analysis**

/* input the data*/

**data** apgar;

input ID y cutpoint t0 t1 t2 t3 t4 t5 t6 t7 t8 t9;

cards;

1 3.534 0 1 0 0 0 0 0 0 0 0 0

2 0 0 1 0 0 0 0 0 0 0 0 0

3 0 0 1 0 0 0 0 0 0 0 0 0

4 0 0 1 0 0 0 0 0 0 0 0 0

5 0 0 1 0 0 0 0 0 0 0 0 0

6 0 0 1 0 0 0 0 0 0 0 0 0

7 0 0 1 0 0 0 0 0 0 0 0 0

8 0 0 1 0 0 0 0 0 0 0 0 0

9 0 0 1 0 0 0 0 0 0 0 0 0

10 0 0 1 0 0 0 0 0 0 0 0 0

11 0 0 1 0 0 0 0 0 0 0 0 0

1 2.621 1 0 1 0 0 0 0 0 0 0 0

2 0 1 0 1 0 0 0 0 0 0 0 0

3 0 1 0 1 0 0 0 0 0 0 0 0

4 0 1 0 1 0 0 0 0 0 0 0 0

5 0 1 0 1 0 0 0 0 0 0 0 0

6 0 1 0 1 0 0 0 0 0 0 0 0

7 0 1 0 1 0 0 0 0 0 0 0 0

8 0 1 0 1 0 0 0 0 0 0 0 0

9 0 1 0 1 0 0 0 0 0 0 0 0

10 0 1 0 1 0 0 0 0 0 0 0 0

11 0 1 0 1 0 0 0 0 0 0 0 0

1 2.67 2 0 0 1 0 0 0 0 0 0 0

2 0 2 0 0 1 0 0 0 0 0 0 0

3 0 2 0 0 1 0 0 0 0 0 0 0

4 0 2 0 0 1 0 0 0 0 0 0 0

5 0 2 0 0 1 0 0 0 0 0 0 0

6 0 2 0 0 1 0 0 0 0 0 0 0

7 0 2 0 0 1 0 0 0 0 0 0 0

8 0 2 0 0 1 0 0 0 0 0 0 0

9 0 2 0 0 1 0 0 0 0 0 0 0

10 0 2 0 0 1 0 0 0 0 0 0 0

11 0 2 0 0 1 0 0 0 0 0 0 0

1 2.599 3 0 0 0 1 0 0 0 0 0 0

2 1.98 3 0 0 0 1 0 0 0 0 0 0

3 2.92 3 0 0 0 1 0 0 0 0 0 0

4 3.265 3 0 0 0 1 0 0 0 0 0 0

5 2.256 3 0 0 0 1 0 0 0 0 0 0

6 1.609 3 0 0 0 1 0 0 0 0 0 0

7 1.314 3 0 0 0 1 0 0 0 0 0 0

8 0 3 0 0 0 1 0 0 0 0 0 0

9 2.311 3 0 0 0 1 0 0 0 0 0 0

10 0.806 3 0 0 0 1 0 0 0 0 0 0

11 0 3 0 0 0 1 0 0 0 0 0 0

1 2.532 4 0 0 0 0 1 0 0 0 0 0

2 0 4 0 0 0 0 1 0 0 0 0 0

3 0 4 0 0 0 0 1 0 0 0 0 0

4 0 4 0 0 0 0 1 0 0 0 0 0

5 0 4 0 0 0 0 1 0 0 0 0 0

6 0 4 0 0 0 0 1 0 0 0 0 0

7 0 4 0 0 0 0 1 0 0 0 0 0

8 3.155 4 0 0 0 0 1 0 0 0 0 0

9 0 4 0 0 0 0 1 0 0 0 0 0

10 0 4 0 0 0 0 1 0 0 0 0 0

11 0 4 0 0 0 0 1 0 0 0 0 0

1 2.501 5 0 0 0 0 0 1 0 0 0 0

2 0 5 0 0 0 0 0 1 0 0 0 0

3 0 5 0 0 0 0 0 1 0 0 0 0

4 0 5 0 0 0 0 0 1 0 0 0 0

5 0 5 0 0 0 0 0 1 0 0 0 0

6 0 5 0 0 0 0 0 1 0 0 0 0

7 0 5 0 0 0 0 0 1 0 0 0 0

8 0 5 0 0 0 0 0 1 0 0 0 0

9 0 5 0 0 0 0 0 1 0 0 0 0

10 0 5 0 0 0 0 0 1 0 0 0 0

11 0 5 0 0 0 0 0 1 0 0 0 0

1 2.383 6 0 0 0 0 0 0 1 0 0 0

2 2.21 6 0 0 0 0 0 0 1 0 0 0

3 2.606 6 0 0 0 0 0 0 1 0 0 0

4 2.997 6 0 0 0 0 0 0 1 0 0 0

5 1.939 6 0 0 0 0 0 0 1 0 0 0

6 0 6 0 0 0 0 0 0 1 0 0 0

7 0 6 0 0 0 0 0 0 1 0 0 0

8 0 6 0 0 0 0 0 0 1 0 0 0

9 0 6 0 0 0 0 0 0 1 0 0 0

10 0 6 0 0 0 0 0 0 1 0 0 0

11 2.386 6 0 0 0 0 0 0 1 0 0 0

1 2.154 7 0 0 0 0 0 0 0 1 0 0

2 0 7 0 0 0 0 0 0 0 1 0 0

3 0 7 0 0 0 0 0 0 0 1 0 0

4 0 7 0 0 0 0 0 0 0 1 0 0

5 0 7 0 0 0 0 0 0 0 1 0 0

6 1.959 7 0 0 0 0 0 0 0 1 0 0

7 0 7 0 0 0 0 0 0 0 1 0 0

8 0 7 0 0 0 0 0 0 0 1 0 0

9 0 7 0 0 0 0 0 0 0 1 0 0

10 0 7 0 0 0 0 0 0 0 1 0 0

11 0 7 0 0 0 0 0 0 0 1 0 0

1 2.021 8 0 0 0 0 0 0 0 0 1 0

2 0 8 0 0 0 0 0 0 0 0 1 0

3 0 8 0 0 0 0 0 0 0 0 1 0

4 0 8 0 0 0 0 0 0 0 0 1 0

5 0 8 0 0 0 0 0 0 0 0 1 0

6 0 8 0 0 0 0 0 0 0 0 1 0

7 0 8 0 0 0 0 0 0 0 0 1 0

8 0 8 0 0 0 0 0 0 0 0 1 0

9 0 8 0 0 0 0 0 0 0 0 1 0

10 0 8 0 0 0 0 0 0 0 0 1 0

11 0 8 0 0 0 0 0 0 0 0 1 0

1 2.111 9 0 0 0 0 0 0 0 0 0 1

2 0 9 0 0 0 0 0 0 0 0 0 1

3 0 9 0 0 0 0 0 0 0 0 0 1

4 0 9 0 0 0 0 0 0 0 0 0 1

5 0 9 0 0 0 0 0 0 0 0 0 1

6 0 9 0 0 0 0 0 0 0 0 0 1

7 0 9 0 0 0 0 0 0 0 0 0 1

8 0 9 0 0 0 0 0 0 0 0 0 1

9 0 9 0 0 0 0 0 0 0 0 0 1

10 0 9 0 0 0 0 0 0 0 0 0 1

11 0 9 0 0 0 0 0 0 0 0 0 1

;

**run**;

/* sort the data */

**Proc** **sort** data=apgar;

by ID cutpoint;

**run**;

/* specify mixed procedure and that ID is a class variable*/

**Proc** **mixed** data=apgar2 method = reml;

class ID ;

/* specify the relationship; here a linear trend is assumed */

/* one could specify any functional relationship of interest here*/

model y = cutpoint / solution cl;

/* specify a random effect on the intercept term */

/* heterogeneity on slope could also be introduced here */

/* specify that the random effect is due to between study heterogeneity */

random int/ subject = ID ;

/* specify that there are multiple estimates from the same study */

/* and enable a seperate variance term for each estimate and a separate */

/* covariance term between each pair of estimates */

repeated / type = un subject = ID group = ID;

/* specify starting values and the within-study variances and covariances*/

parms

/* the between-study variance on the intercept starting value */

**0.01**

/* the within-study variance-covariance matrix for study 1*/

**0.306916**

**0.014138**

**0.026569**

**0.01223**

**0.016215**

**0.019881**

**0.00712**

**0.013131**

**0.016464**

**0.018496**

**0.006179**

**0.010667**

**0.013731**

**0.015602**

**0.018769**

**0.005412**

**0.008966**

**0.011719**

**0.013544**

**0.016127**

**0.020736**

**0.004866**

**0.007876**

**0.010481**

**0.012259**

**0.014735**

**0.018803**

**0.023409**

**0.004048**

**0.007308**

**0.009916**

**0.011507**

**0.01372**

**0.017451**

**0.021746**

**0.027889**

**0.004202**

**0.006053**

**0.008169**

**0.009555**

**0.011652**

**0.015428**

**0.019503**

**0.024924**

**0.053361**

**0.002036**

**0.003125**

**0.004275**

**0.005738**

**0.007285**

**0.009302**

**0.011727**

**0.01479**

**0.033873**

**0.259081**

/* study 2 */

**1.00E+06**

**0**

**1.00E+06**

**0**

**0**

**1.00E+06**

**0**

**0**

**0**

**0.038809**

**0**

**0**

**0**

**0**

**1.00E+06**

**0**

**0**

**0**

**0**

**0**

**1.00E+06**

**0**

**0**

**0**

**0.027039**

**0**

**0**

**0.090601**

**0**

**0**

**0**

**0**

**0**

**0**

**0**

**1.00E+06**

**0**

**0**

**0**

**0**

**0**

**0**

**0**

**0**

**1.00E+06**

**0**

**0**

**0**

**0**

**0**

**0**

**0**

**0**

**0**

**1.00E+06**

/* study 3*/

**1.00E+06**

**0**

...

/* and so on for all studies */

/* state that the first term is to be estimated, but that all the with-study */

/* covariance matrices are fixed and known */

/ eqcons = **2** to **606**;

/* estimate the log odds ratio at each cut-point based on the model estimated */

estimate 'logor0' int **1** / cl df = **1000**;

estimate 'logor1' int **1** cutpoint **1** / cl df = **1000**;

estimate 'logor2' int **1** cutpoint **2** / cl df = **1000**;

estimate 'logor3' int **1** cutpoint **3** / cl df = **1000**;

estimate 'logor4' int **1** cutpoint **4** / cl df = **1000**;

estimate 'logor5' int **1** cutpoint **5** / cl df = **1000**;

estimate 'logor6' int **1** cutpoint **6** / cl df = **1000**;

estimate 'logor7' int **1** cutpoint **7** / cl df = **1000**;

estimate 'logor8' int **1** cutpoint **8** / cl df = **1000**;

estimate 'logor9' int **1** cutpoint **9** / cl df = **1000**;

**run**;

**Supplementary Material S2:** Full Apgar data

| Study | Cut-point | Number with Apgar score less than or equal to the cut-point & dead | Number with Apgar score less than or equal to the cut-point & alive | Number with Apgar score greater than the cut-point & dead | Number with Apgar score greater than the cut-point & alive | Total |
| --- | --- | --- | --- | --- | --- | --- |
| Apgar | 0 | 19 | 4 | 292 | 2107 | 2422 |
| Apgar | 1 | 112 | 83 | 199 | 2028 | 2422 |
| Apgar | 2 | 167 | 157 | 144 | 1954 | 2422 |
| Apgar | 3 | 189 | 218 | 122 | 1893 | 2422 |
| Apgar | 4 | 213 | 311 | 98 | 1800 | 2422 |
| Apgar | 5 | 237 | 439 | 74 | 1672 | 2422 |
| Apgar | 6 | 253 | 606 | 58 | 1505 | 2422 |
| Apgar | 7 | 266 | 859 | 45 | 1252 | 2422 |
| Apgar | 8 | 290 | 1365 | 21 | 746 | 2422 |
| Apgar | 9 | 307 | 1906 | 4 | 205 | 2422 |
| Beeby | 3 | 49 | 135 | 39 | 400 | 623 |
| Behnke | 3 | 113 | 144 | 48 | 443 | 748 |
| Behnke | 6 | 148 | 326 | 13 | 261 | 748 |
| Drage | 3 | 101 | 144 | 50 | 1322 | 1617 |
| Drage | 6 | 128 | 427 | 23 | 1039 | 1617 |
| Heller | 3 | 97 | 1267 | 91 | 31106 | 32561 |
| Heller | 6 | 147 | 4916 | 41 | 27457 | 32561 |
| Ikonen | 3 | 35 | 25 | 65 | 443 | 568 |
| Ikonen | 6 | 55 | 70 | 45 | 398 | 568 |
| Issel | 3 | 20 | 45 | 52 | 585 | 702 |
| Issel | 7 | 52 | 169 | 20 | 461 | 702 |
| Kato | 4 | 6 | 75 | 0 | 147 | 228 |
| Luthy | 3 | 26 | 47 | 9 | 164 | 246 |
| Serenius | 3 | 28 | 30 | 45 | 108 | 211 |
| Tejani | 6 | 47 | 142 | 6 | 197 | 392 |
